# From Mimicry to Language: A Neuroanatomically Based Evolutionary Model of the Emergence of Vocal Language

**DOI:** 10.3389/fnins.2016.00307

**Published:** 2016-06-30

**Authors:** Oren Poliva

**Affiliations:** Department of Psychology, Bangor UniversityBangor, UK

**Keywords:** language, speech, aphasia, auditory dorsal stream, auditory ventral stream, evolution

## Abstract

The auditory cortex communicates with the frontal lobe via the middle temporal gyrus (auditory ventral stream; AVS) or the inferior parietal lobule (auditory dorsal stream; ADS). Whereas the AVS is ascribed only with sound recognition, the ADS is ascribed with sound localization, voice detection, prosodic perception/production, lip-speech integration, phoneme discrimination, articulation, repetition, phonological long-term memory and working memory. Previously, I interpreted the juxtaposition of sound localization, voice detection, audio-visual integration and prosodic analysis, as evidence that the behavioral precursor to human speech is the exchange of contact calls in non-human primates. Herein, I interpret the remaining ADS functions as evidence of additional stages in language evolution. According to this model, the role of the ADS in vocal control enabled early *Homo* (*Hominans*) to name objects using monosyllabic calls, and allowed children to learn their parents' calls by imitating their lip movements. Initially, the calls were forgotten quickly but gradually were remembered for longer periods. Once the representations of the calls became permanent, mimicry was limited to infancy, and older individuals encoded in the ADS a lexicon for the names of objects (phonological lexicon). Consequently, sound recognition in the AVS was sufficient for activating the phonological representations in the ADS and mimicry became independent of lip-reading. Later, by developing inhibitory connections between acoustic-syllabic representations in the AVS and phonological representations of subsequent syllables in the ADS, *Hominans* became capable of concatenating the monosyllabic calls for repeating polysyllabic words (i.e., developed working memory). Finally, due to strengthening of connections between phonological representations in the ADS, *Hominans* became capable of encoding several syllables as a single representation (chunking). Consequently, *Hominans* began vocalizing and mimicking/rehearsing lists of words (sentences).

## Introduction

In his seminal book *The Descent of Man*, Darwin (1871) proposed that language emerged from the perception and production of musical performances during mating rituals. More recently, scholars have also proposed that early members of the genus *Homo* (i.e., *Hominans*; Wood and Richmond, 2000) sang rather than talked to each other (Mithen, 2006) and that these songs were the precursor to human language. Other scholars have proposed that early *Hominans* communicated via hand gestures in a language similar to contemporary sign languages. Only after this gestural language developed grammatical rules did language become vocal (Studdert-Kennedy, 1970; Hewes, 1973; Donald, 2005; Gentilucci and Corballis, 2006; Arbib, 2008; Corballis, 2010). One controversial model has even proposed that because the use of grammar provides no evolutionary advantage, a mutation in a mechanism for navigation, social interaction or arithmetical thinking may have resulted in the abrupt emergence of language in its final form (Chomsky, 1986; Hauser et al., 2002; although see Pinker and Jackendoff, 2005 for counter-arguments).

Recently, I have proposed a novel evolutionary account of the emergence of the first conversation (the “From Where To What” model; Poliva, 2015). In this model, the behavioral precursor to present-day speech in non-human primates is the exchange of calls that are used by mothers and their offspring to determine one another's location in cases of separation (i.e., contact calls). As the *Homo* genus emerged, early *Hominans* (e.g., *Homo habilis*) became capable of modifying these calls with intonations. During separation, infants became capable of signaling to their mothers whether they were experiencing low or high levels of distress. This ability to use intonations eventuated the first question and answer conversation. In this scenario, an infant emitted a low-level distress call to signal its desire to interact with an object. The mother then responded with a low-level distress call to signal approval or a high-level distress call to discourage the interaction. As generations passed, the prevalent use of intonations resulted in later *Hominans* acquiring incrementally more volitional control over the vocal apparatus. Eventually, the ability to use intonations to modify calls developed into speech as individuals became capable of associating objects with their own unique calls (i.e., proto-words).

In the model proposed in this study, I provide a novel account of the emergence of present-day language. The model describes the period after *Hominans* acquired volitional control over the vocal apparatus and can thus be considered a direct continuation of the “From Where To What” model. This model describes four chapters in our evolutionary story: (1) After developing volitional vocal control, adult individuals began inventing calls and associating them with objects (i.e., proto-words), and their offspring learned these proto-words by mimicking their parents. This mimicry marked a transition from offspring inquiring about the safety of interacting with objects (proposed in Poliva, 2015) to children inquiring about the names of objects. Initially, this mimicry was dependent on the child intently focusing on his/her parent's lip movements and imitating them. This dependence on observing lip movements may be the reason that present-day humans have much more conspicuous lips than any of our apian relatives. These learned calls were short (monosyllabic) and were forgotten soon after they were learned. (2) Over generations, the representations of the calls, which were encoded in the posterior temporal-parietal region, became incrementally more robust. Consequently, the calls could be remembered for increasing lengths of time. Eventually, the representations of the calls in the posterior temporal-parietal region became immune to decay and began to be remembered after the first encounter during infancy. Because the mimicked calls were encoded in both a pre-existing long-term memory store through sound recognition (located in the middle temporal gyrus) and in the new long-term vocal memory store (located in the posterior temporal-parietal region), the practice of mimicry led to the formation of associations between related representations in the two memory stores. Consequently, though infants still mimicked their parents by imitating lip movements, older children became capable of mimicking calls through sound recognition, without observing lip movements. This development enabled parents to teach calls at night and in caves, when more time was available for practice. Because of this development, present-day infants constantly mimic their parents' vocalizations. (3) Because a vocabulary of monosyllabic calls is necessarily limited, words with increasing numbers of syllables were created by concatenating the monosyllabic calls. The repetition of these polysyllabic calls by their offspring led to gradual development of verbal working memory. (4) In the final stage, through rehearsal, individuals became capable of encoding several syllables as a single word (i.e., chunking). The emergence of chunking enabled individuals to rehearse lists of words, instead of syllables, in working memory and consequently to communicate these lists to others. These word lists were the first sentences.

## Neuroanatomy of language

**Figure 1:**
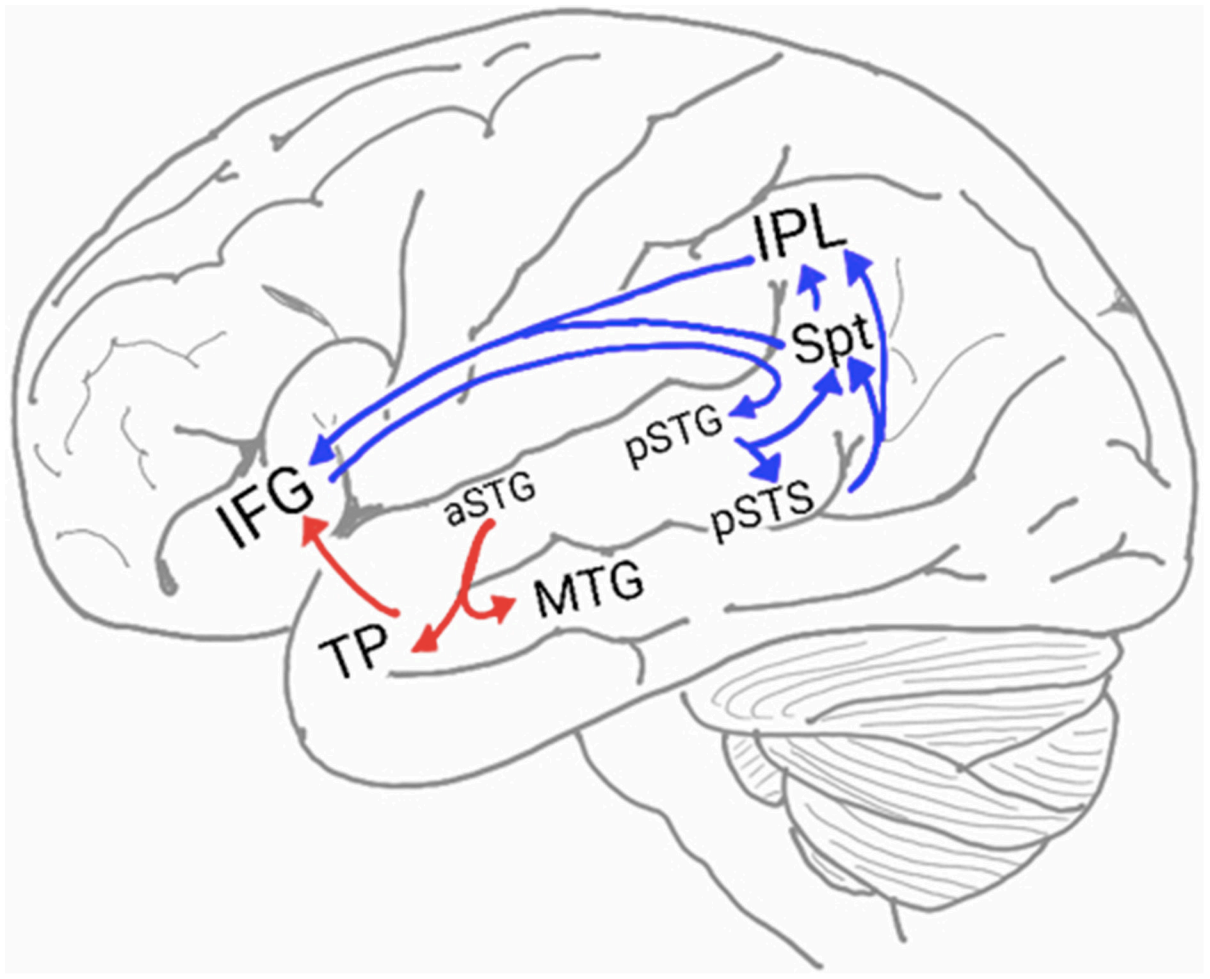
**The neuroanatomy of the auditory ventral and dorsal streams**. Two pathways connect the auditory cortex and the inferior frontal gyrus (IFG): the auditory ventral stream (AVS; red arrows), which processes sound recognition, and the auditory dorsal stream (ADS; blue arrows), which processes sound localization, speech production and repetition, phonological working memory, phonological long-term memory and more. In the AVS, the anterior superior temporal gyrus (aSTG) communicates with the IFG via relay stations in the middle temporal gyrus (MTG) and temporal pole (TP). In the ADS, the posterior superior temporal gyrus (pSTG) communicates with the IFG via relay stations in the posterior superior temporal sulcus (pSTS), Sylvian parietal-temporal junction (Spt) and inferior parietal lobule (IPL).

In humans, two pathways connect the auditory cortex and the frontal lobe (Figure 1; Hickok and Poeppel, 2007; Rauschecker and Scott, 2009; Gow, 2012; Poliva, 2015). The first pathway, the auditory ventral stream (AVS), connects the anterior auditory cortex (aSTG) with the inferior frontal gyrus (IFG) in the frontal lobe via relay stations in the middle temporal gyrus (MTG) and temporal pole (TP). The second pathway, the auditory dorsal stream (ADS), connects the posterior auditory cortex (pSTG) with several frontal lobe regions (including the IFG) via relay stations in the posterior superior temporal sulcus (pSTS), Sylvian parieto-temporal junction (Spt) and inferior parietal lobule (IPL).

The AVS is commonly associated with the role of sound recognition and is often referred to as the auditory “What” pathway (Hickok and Poeppel, 2007; Poliva, 2015). Sound recognition occurs in two stages: first, the aSTG recognizes the acoustic pattern (Scott et al., 2000; Davis and Johnsrude, 2003; Poliva, 2015), and downstream, the MTG and TP match the sound with its corresponding audio-visual semantic representation from long-term memory (i.e., the semantic lexicon; Patterson et al., 2007; Gow, 2012). This recognition of sounds in the AVS, although critical for effective communication, appears to contribute less to the uniqueness of human language than the ADS. This is evident in the universality of sound recognition, which many mammalian species use to identify prey, predators or potential mates. For example, fMRI studies have shown that the ability of dogs to recognize spoken words and extract their meaning (Kaminski, 2004; Pilley and Reid, 2011) is localized in the TP of the AVS (Andics et al., 2014). Apes trained in human facilities have also been reported to be capable of learning human speech and comprehending its meaning. For example, it has been reported that the bonobos Kanzi and Panbanisha can recognize more than 3000 spoken English words (Blake, 2004; Gibson, 2011).

In contrast to the relatively preserved function of the AVS in mammals, the ADS has been associated with a broad range of functions. The most established function of the ADS is sound localization, and, appropriately, this processing stream is often referred to as the auditory “Where” pathway (Clarke et al., 2000; Tian et al., 2001). In addition to sound localization, ADS regions (pSTG, pSTS, Spt, IPL, IFG) have been ascribed with a broad range of functions, including discrimination/ identification of speakers (Lachaux et al., 2007; Jardri et al., 2012), prosodic perception and expression (Hickok et al., 2003), audio-visual integration (with emphasis on lip-reading; Nishitani and Hari, 2002; Campbell, 2008; Kayser et al., 2009), phoneme discrimination (Turkeltaub and Coslett, 2010), object naming (Schwartz et al., 2012; Roux et al., 2015), speech repetition and articulation (Warren et al., 2005; Hickok and Poeppel, 2007), phonological working memory (Buchsbaum and D'Esposito, 2008) and phonological long-term memory (Gow, 2012). Given this diversity, it is unlikely that the ADS is responsible for a single computation that is shared among all these functions (e.g., it is difficult to describe a common computation between sound localization and lip-reading). Moreover, as most of these functions were localized to two or all ADS regions, it is also unlikely that this functional co-localization is a mere coincidence. In the present paper, I propose that the function of ADS changed and modified as language evolved. Hence, the functions of the ADS are vestigial and thus provide us with clues to the nature of intermediate stages in the development of language. Corroborating the involvement of the ADS in the development of language is a study that reconstructed the endocranium of early *Hominins*. The results showed that *Homo habilis*, but not any of its *Australopith* ancestors, is characterized by a dramatic heightening of the IPL and an enlargement (though to a lesser degree) of the IFG, whereas the rest of its endocranium remains highly similar to the endocranium of modern apes (Tobias, 1987). Further consistent with the role of the ADS in language evolution, a diffusion tensor imaging study that compared the white matter of humans to that of chimpanzees reported significantly stronger connectivity in the human ADS but not in the human AVS (Rilling et al., 2011).

In my previous model (Poliva, 2015), I interpreted the involvement of the ADS in sound localization, voice detection and face-call integration as evidence that the role of the ADS in non-human primates is the detection of contact calls and that, via connections with the brainstem, this processing stream also mediates the emission of these calls. Moreover, I have proposed that the contribution of the ADS to the perception and production of intonations (prosody) is evidence that modifications to the ADS and its connections with the brainstem endowed our *Hominan* ancestors with partial vocal control. In the remaining sections of this paper, I present detailed evidence for the remaining functions of the ADS and interpret their juxtaposition in the ADS as evidence of additional forgotten chapters in our language evolution story.

### The ADS and vocal mimicry

In my previous model, I proposed that, owing to changes in the ADS of early *Hominans*, mothers and children were capable of interacting in a vocal manner resembling conversation. In this scenario, children emitted low-level distress calls to alert their mothers that they were interested in exploring an object. The mothers then responded with a low- or high-level distress call to signal approval or disapproval, respectively. Such proto-conversations, however, are limited in content because the meaning of each call is dependent on the context. For speech to become more versatile, early *Hominans* needed a method for acquiring vocabulary. A possible route for the acquisition of words is that the prevalence of using intonations gradually resulted in an increase in volitional control over the vocal apparatus. Eventually, *Hominans* developed sufficient vocal control to invent novel calls, and offspring began mimicking their parents.

**Figure 2:**
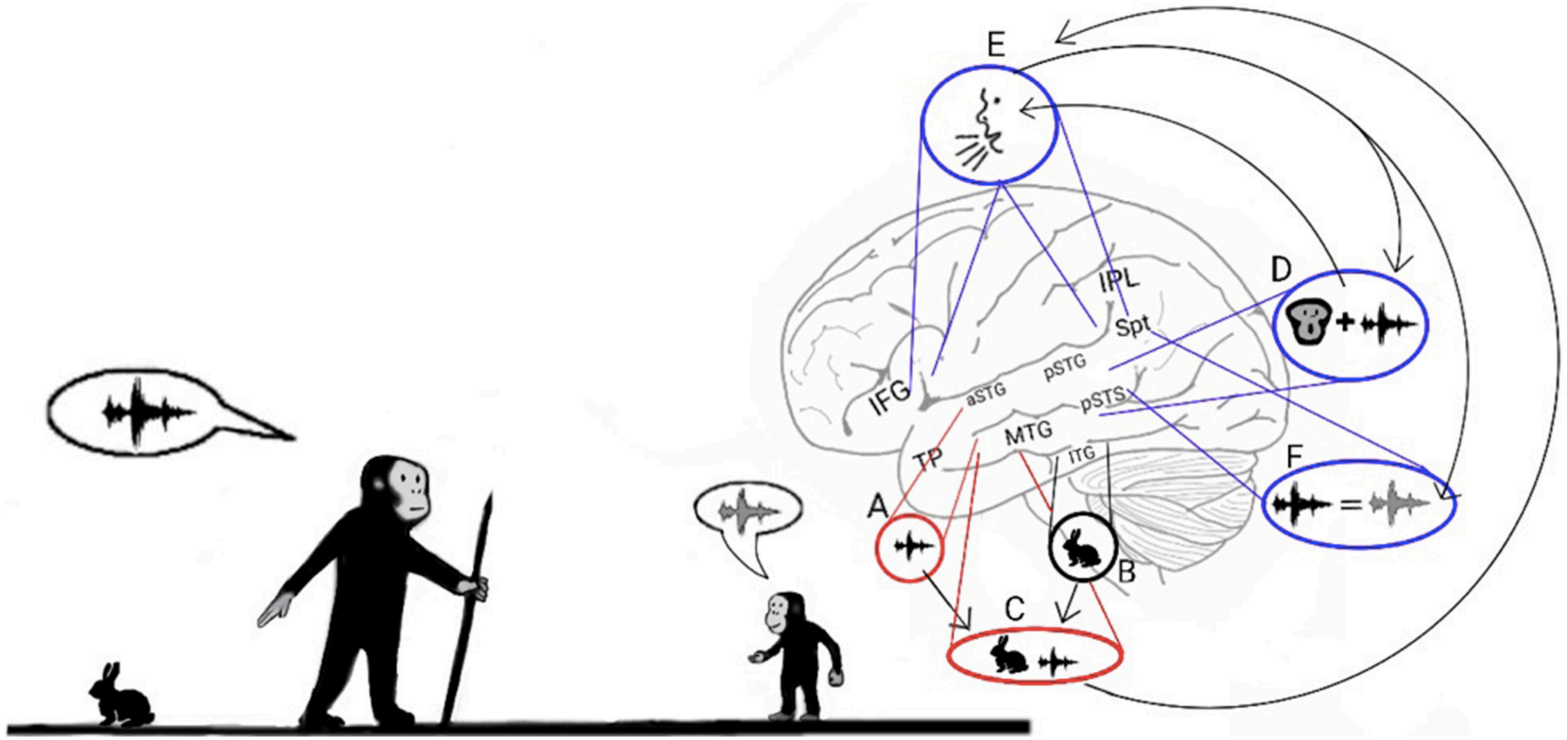
**Stage 1: Mimicry and the imitation of lip-movements**. The model proposes that early *Hominans* evolved to mimic vocalizations by reading lips to learn novel words. Here, an adult teaches a child the vocalization associated with a rabbit (Left). The adult recognizes the rabbit through auditory object recognition in the aSTG **(A)** or through visual object recognition in the inferior temporal gyrus (ITG; **B**) and associates it with the concept of a rabbit that is coded in the semantic lexicon of the MTG-TP **(C)**. The adult vocalizes the call associated with a rabbit by projecting from the semantic representation in the MTG to the praxic representations in area Spt and then to the IFG **(E)**. The child learns the call associated with the animal by repeating the call. When the child hears the call, he/she encodes the acoustic properties of the call in the sound recognition center of the aSTG **(A)** and learns to associate the call with its semantic representation **(C)**. In parallel, the pSTG-pSTS receive their own afferents from the auditory cortex and extract phonemic information from it. Via processing in the pSTG-pSTS of the ADS, the child then integrates the phoneme with its corresponding lip movements **(D)**. The pSTG-pSTS then activate the praxic representation of the call in area Spt and then in the IFG **(E)**. Finally, via feedback connections from the IFG to the Spt-pSTG, the child recognizes the emitted call as self-produced and verifies that the emitted call is acoustically similar to the call that he/she previously perceived **(F)**. The child repeats this process until he/she can vocalize the call associated with a rabbit on his/her own.

Consistent with the previous model, which ascribes the production of distress calls to ADS processing, studies of present-day humans have demonstrated the ADS' role in speech production, particularly in the vocal expression of the names of objects. For instance, in a series of studies in which sub-cortical fibers were directly stimulated (Duffau, 2008), interference in the left pSTG and IPL resulted in errors during object-naming tasks, and interference in the left IFG resulted in speech arrest. Magnetic interference in the pSTG and IFG of healthy participants also produced speech errors and speech arrest, respectively (Stewart et al., 2001; Acheson et al., 2011). One study has also reported that electrical stimulation of the left IPL caused patients to believe that they had spoken when they had not and that IFG stimulation caused patients to unconsciously move their lips (Desmurget et al., 2009). The contribution of the ADS to the process of articulating the names of objects appears to be dependent on the reception of afferents from the semantic lexicon of the AVS (Figure 2—arrow between **C** and **E**), as evidenced by an intra-cortical recording study that reported activation in the posterior MTG prior to activation in the Spt-IPL region when patients named objects in pictures (Edwards et al., 2010). Additional evidence has been shown in intra-cortical electrical stimulation studies in which interference to the posterior MTG was correlated with impaired object naming (Boatman et al., 2000; Matsumoto et al., 2011).

In addition to speech production, the ADS is also associated with several aspects of speech perception. The role of the ADS in processing spoken words is evident in a meta-analysis of fMRI studies in which the auditory perception of phonemes was contrasted with closely matching sounds (Turkeltaub and Coslett, 2010). The authors divided these studies into those requiring high and low levels of attention to phonemes and concluded that attention to phonemes correlates with strong activation in the pSTG-pSTS region. An intra-cortical recording study in which participants were instructed to identify syllables also correlated the hearing of each syllable with its own activation pattern in the pSTG (Chang et al., 2010). The involvement of the ADS in both speech perception and production has been further illuminated in several pioneering functional imaging studies that contrasted speech perception with overt or covert speech production (Buchsbaum et al., 2001; Wise et al., 2001; Hickok et al., 2003). These studies demonstrated that the pSTS is active only during the perception of speech, whereas area Spt is active during both the perception and production of speech. The authors concluded that the pSTS projects to area Spt, which converts the auditory input into articulatory movements (Warren et al., 2005; Hickok and Poeppel, 2007). Similar results have been obtained in a study in which participants' temporal and parietal lobes were electrically stimulated. This study reported that electrically stimulating the pSTG region interferes with sentence comprehension and that stimulation of the IPL interferes with the ability to vocalize the names of objects (Roux et al., 2015). The authors also reported that stimulation in area Spt and the inferior IPL induced interference during both object-naming and speech-comprehension tasks. The role of the ADS in speech repetition is also congruent with the results of the other functional imaging studies that have localized activation during speech repetition tasks to ADS regions (Karbe et al., 1998; Giraud and Price, 2001; Graves et al., 2008). An intra-cortical recording study that recorded activity throughout most of the temporal, parietal and frontal lobes also reported activation in the pSTG, Spt, IPL and IFG when speech repetition is contrasted with speech perception (Towle et al., 2008). Neuropsychological studies have also found that individuals with speech repetition deficits but preserved auditory comprehension (i.e., conduction aphasia) suffer from circumscribed damage to the Spt-IPL area (Selnes et al., 1985; Axer et al., 2001; Bartha and Benke, 2003; Baldo et al., 2008, 2012; Fridriksson et al., 2010; Buchsbaum et al., 2011) or damage to the projections that emanate from this area and target the frontal lobe (Yamada et al., 2007; Breier et al., 2008; Zhang et al., 2010; Parker Jones et al., 2014). Studies have also reported a transient speech repetition deficit in patients after direct intra-cortical electrical stimulation to this same region (Anderson et al., 1999; Quigg and Fountain, 1999; Quigg et al., 2006). Insight into the purpose of speech repetition in the ADS is provided by longitudinal studies of children that correlated the learning of foreign vocabulary with the ability to repeat nonsense words (Service, 1992; Service and Kohonen, 1995). In the present model, the role of the ADS in speech production and repetition suggests that soon after early *Hominans* began to associate vocalizations with objects through MTG-Spt-IFG connections, children became capable of learning these calls by mimicking them through pSTG/pSTS-Spt-IFG connections. Rare reports of brain-damaged aphasic patients with compulsive speech repetition (echolalia; Geschwind et al., 1968; Fay and Colleman, 1977; Bogousslavsky et al., 1988; Rapcsak et al., 1990; Mendez, 2002) further suggest that the repetition of early *Hominans* was automatic and uncontrollable.

### The ADS and the imitation of lip movements

The evidence presented so far supports the hypothesis that the ADS was modified during *Hominan* evolution to enable adults to teach their children words through mimicry. Mimicking, however, is a complex process, and to accomplish it, children must first be familiar with the relationship between the shapes of their mouths and the sounds they emit. Hence, I propose that the first vocal mimicry involved a child intently inspecting his or her parents' lip movements, imitating the lip movements, and then comparing the produced call to the heard call (Figure 2). This dependence on lip reading for novel word acquisition was likely similar to the imitation of lip movements that occurs today when adult individuals learn to pronounce foreign words (Wagner, 2007) and was thus a laborious process.

Consistent with the role of the ADS in discriminating phonemes (Turkeltaub and Coslett, 2010), studies have ascribed the integration of phonemes and their corresponding lip movements (i.e., visemes) to the pSTS of the ADS. For example, an fMRI study (Nath and Beauchamp, 2012) has correlated activation in the pSTS with the McGurk illusion (in which hearing the syllable “ba” while seeing the viseme “ga” results in the perception of the syllable “da”). Another study has found that using magnetic stimulation to interfere with processing in this area further disrupts the McGurk illusion (Beauchamp et al., 2010). The association of the pSTS with the audio-visual integration of speech has also been demonstrated in a study that presented participants with pictures of faces and spoken words of varying quality. The study reported that the pSTS selects for the combined increase of the clarity of faces and spoken words (McGettigan et al., 2012). Corroborating evidence has been provided by an fMRI study that contrasted the perception of audio-visual speech with audio-visual non-speech (pictures and sounds of tools; Stevenson and James, 2009). This study reported the detection of speech-selective compartments in the pSTS. In addition, an fMRI study that contrasted congruent audio-visual speech with incongruent speech (pictures of still faces) reported pSTS activation (Bernstein et al., 2010) (for a review presenting additional converging evidence regarding the role of the pSTS and ADS in phoneme-viseme integration see Campbell, 2008).

A growing body of evidence corroborates the hypothesis that the analysis of lip movements was critical to the development of vocal mimicry. Studies have shown that when people articulate a syllable while viewing another person articulating the same syllable, they are better at identifying the syllable (Sams et al., 2005) and vocalize it with a shorter reaction time (Kerzel and Bekkering, 2000) than when they watch another person articulating a different syllable. The influence of lip movements on mimicry has also been demonstrated in a study that requested participants to mimic heard syllables while perceiving incongruent visemes (i.e., the McGurk illusion; Gentilucci and Cattaneo, 2005). This study reported that individuals modified their emitted syllable to be more similar to the perceived syllable only when the perceived viseme was easily discernible (labial) and the viseme associated with the perceived phoneme wasn't (alveolar, velar). An MEG study in which participants were instructed to observe or imitate a series of pictures of lip movements or to spontaneously generate lip movements on their own reported the spreading of activation from the occipital lobe to the ADS (occipital lobe to the pSTS, IPL, IFG and then to the primary motor cortex) only in the observation and imitation conditions (Nishitani and Hari, 2002). The activation in the ADS was also much stronger during the imitation condition than during the observation condition. Further supporting the involvement of the ADS in integrating speech with visemes in the mimicry process is the finding that conduction aphasia patients with either temporal or parietal lobe lesions demonstrate impairment during tasks in which they must imitate sequences of lip-movements (Kimura and Watson, 1989). The inverse relationship between phonemic and visemic similarity (e.g., the phonemes “m” and “n” and “th” and “f” are acoustically similar but visually distinctive; in contrast, the phonemes “p” and “b” are acoustically distinct, but visually similar; Summerfield, 1987) also supports the theory of the co-development of phonemes and visemes in the early stages of language evolution. The hypothesis that lip-reading was critical to the emergence of language is also supported by the perception of full lips as an attractive sexual quality in present-day humans (especially in females; Michiels and Sather, 1994; Bisson and Grobbelaar, 2004) and the resulting universal *Homo sapiens* phenotype of conspicuously visible and protruding lips, which has not been observed in other apes. This finding implies that not only did lip movements undergo evolutionary modification to facilitate speech imitation, but lip shape and color were also modified.

### The ADS and voice monitoring

In the present model, I propose that the children of early *Hominans* learned new words by focusing on their parents' lip movements, imitating the same movements, emitting a call, and then comparing the emitted call to the heard call. I have already demonstrated that the ADS is involved in the imitation of lip-movements and vocal mimicry but have not provided evidence of its role in the monitoring of vocalizations. Neuroanatomical evidence suggests that the ADS is equipped with descending connections from the IFG to the pSTG that relay information about motor activity (i.e., corollary discharges) in the vocal apparatus (mouth, tongue, vocal folds). This feedback marks the sound perceived during speech as self-produced and can thus be used to adjust the vocal apparatus to increase the similarity between the perceived and emitted calls. Evidence for descending connections from the IFG to the pSTG has been offered by a study that electrically stimulated the IFG during surgical operations and reported the spread of activation to the pSTG-pSTS-Spt region (Matsumoto, 2004). A study (Kimura and Watson, 1989) that compared the ability of aphasic patients with frontal, parietal or temporal lobe damage to quickly and repeatedly articulate a string of syllables reported that damage to the frontal lobe interfered with the articulation of both identical syllabic strings (“Bababa”) and non-identical syllabic strings (“Badaga”), whereas patients with temporal or parietal lobe damage only exhibited impairment when articulating non-identical syllabic strings. Because the patients with temporal and parietal lobe damage were capable of repeating the syllabic string in the first task, their speech perception and production appears to be relatively preserved, and their deficit in the second task is therefore due to impaired monitoring. Demonstrating the role of the descending ADS connections in monitoring emitted calls, an fMRI study instructed participants to speak under normal conditions or when hearing a modified version of their own voice (delayed first formant) and reported that hearing a distorted version of one's own voice results in increased activation in the pSTG (Tourville et al., 2008). Further demonstrating that the ADS facilitates motor feedback during mimicry is an intra-cortical recording study that contrasted speech perception and repetition (Towle et al., 2008). The authors reported that, in addition to activation in the IPL and IFG, speech repetition is characterized by stronger activation in the pSTG than during speech perception. (for additional converging evidence regarding the role of the ADS in the relay of feedback motor connections from the vocal apparatus, see Rauschecker and Scott, 2009; Rauschecker, 2011).

### The ADS and the phonological lexicon

Early *Hominans*' ability to vocally name objects likely evolved gradually. Early in the evolutionary process, the neural trace of the calls decayed quickly. However, due to selective pressures that favored individuals with more robust representations of calls in the ADS, the neural trace of the calls began to last for longer periods. Eventually, the neural trace of these calls became immune to decay, and the learned calls became permanent. In present-day humans, the long-term encoding of these representations is called the phonological lexicon.

A growing body of evidence indicates that humans, in addition to having a long-term store for word meanings located in the MTG-TP of the AVS (i.e., the semantic lexicon), also have a long-term store for the names of objects located in the Spt-IPL region of the ADS (i.e., the phonological lexicon). For example, a study (Schwartz et al., 2009, 2012) examining patients with damage to the AVS (MTG damage) or damage to the ADS (IPL damage) reported that MTG damage results in individuals incorrectly identifying objects (e.g., calling a “goat” a “sheep,” an example of semantic paraphasia). Conversely, IPL damage results in individuals correctly identifying the object but incorrectly pronouncing its name (e.g., saying “gof” instead of “goat,” an example of phonemic paraphasia). Semantic paraphasia errors have also been reported in patients receiving intra-cortical electrical stimulation of the AVS (MTG), and phonemic paraphasia errors have been reported in patients whose ADS (pSTG, Spt, and IPL) received intra-cortical electrical stimulation (Ojemann, 1983; Duffau, 2008; Roux et al., 2015). Further supporting the role of the ADS in object naming is an MEG study that localized activity in the IPL during the learning and during the recall of object names (Cornelissen et al., 2004). Similarly, an fMRI study (Breitenstein et al., 2005) has demonstrated that activation increases in the IPL, inferior-temporal gyrus (responsible for visual object recognition) and hippocampus (the memory formation center) of participants learning to associate objects with nonsense words. A study that induced magnetic interference in participants' IPL while they answered questions about an object reported that the participants were capable of answering questions regarding the object's characteristics or perceptual attributes but were impaired when asked whether the word contained two or three syllables (Hartwigsen et al., 2010). An MEG study has also correlated recovery from anomia (a disorder characterized by an impaired ability to name objects) with changes in IPL activation (Cornelissen et al., 2003). Further supporting the role of the IPL in encoding the sounds of words are studies reporting that, compared to monolinguals, bilinguals have greater cortical density in the IPL but not the MTG (Mechelli et al., 2004; Green et al., 2007). Because evidence shows that, in bilinguals, different phonological representations of the same word share the same semantic representation (Francis, 2005-review), this increase in density in the IPL verifies the existence of the phonological lexicon: the semantic lexicon of bilinguals is expected to be similar in size to the semantic lexicon of monolinguals, whereas their phonological lexicon should be twice the size. Consistent with this finding, cortical density in the IPL of monolinguals also correlates with vocabulary size (Lee et al., 2007; Richardson et al., 2010). Notably, the functional dissociation of the AVS and ADS in object-naming tasks is supported by cumulative evidence from reading research showing that semantic errors are correlated with MTG impairment and phonemic errors with IPL impairment. Based on these associations, the semantic analysis of text has been linked to the inferior-temporal gyrus and MTG, and the phonological analysis of text has been linked to the pSTG-Spt-IPL (Jobard et al., 2003; Bolger et al., 2005; Spitsyna et al., 2006; Brambati et al., 2009). The similarity between the symptoms that occur after impairment to the MTG and IPL in both reading and object naming implies that the recently acquired ability to read text evolved from the ability to name visual objects.

### Vocal mimicry and audio-visual integration in infancy

**Figure 3:**
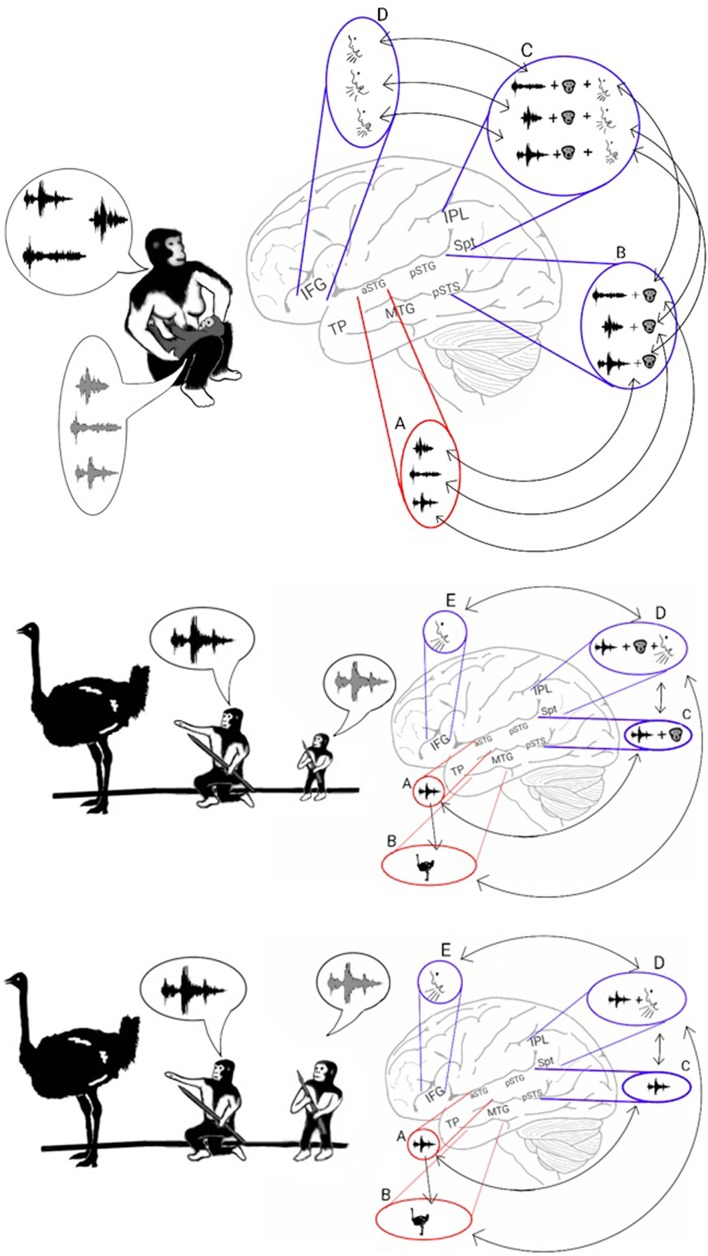
**Stage 2: Vocal mimicry at infancy and the liberation from imitating lip-movements**. Top: As the infant mimicked each of the parents' calls, the acoustic properties of the call were encoded in the aSTG **(A)**. In parallel, the phonemic and visemic representations of the call **(B)** were integrated with its praxic representation in area Spt **(C)**. The infant then produced the call by projecting to the motor regions of the IFG **(D)**. The long-term memory store for the vocal properties of calls in the Spt-IPL region is called the phonological lexicon. As during mimicry, sound recognition and the integration of phonemes with their visemes occur at the same time, associations formed between the acoustic representations of the aSTG and the corresponding phonological representations of the ADS (arrows between **A** and **B**). Middle: Individuals, who passed the vocal mimicry stage during infancy, also recognized sounds via the aSTG **(A)** and extracted its meaning via the MTG-TP **(B)**. Given the presence of phonemic-visemic representations in pSTG that are associated with the perceived sound, individuals were capable of mimicking the call via aSTG to pSTG connections (arrow between **A** and **C**). Moreover, given the presence of phonemic-visemic-praxic (phonological) representations in the Spt-IPL region, individuals were capable of naming the object they see/hear via MTG-TP to Spt-IPL connections (arrow between **B** and **D**). Activation of the phonological representation then activated the motor cortex in the IFG, which initiated the vocalization of the word **(E)**. Bottom: After years of practicing the word without relying on lip movement imitation, the connections with the visemic representations become less robust, and the phonological representations eventually came to comprise primarily the phonemic and praxic representations.

An interesting secondary effect of achieving the permanent encoding of phonological representations in the phonological lexicon is that the learning of these representations occurs only when they are first introduced during infancy. Therefore, practicing vocal mimicry by observing lip movements is restricted to this developmental period (Figure 3, top). Cumulative evidence corroborates the emergence of a brief period during which infants constantly mimic calls by integrating visemic and acoustic speech properties. The description presented here of a critical period for language acquisition is consistent with results from numerous studies reporting that present-day infants between the ages of 6 and 12 months acquire the ability to enunciate the phonemes that are unique to their language through vocal mimicry, whereas learning to pronounce such phonemes at a later age is considerably more difficult (Kuhl, 2004-review). Studies have also shown that during this critical period, present-day infants integrate acoustic and visemic information when learning to speak. This process has been demonstrated by eye-tracking experiments reporting that healthy 9- to 12-month-old infants looked at their mother's lips when listening to her speak with greater frequency than 6-month-old infants did (Tenenbaum et al., 2012). Furthermore, the 12-month-old infants stared at their mother's lips for a longer duration when she spoke in an unfamiliar language, evidence of speech-related learning (Kubicek et al., 2013). Evidence that infants process both speech and lip movements is also shown in a study that habituated infants to seeing and hearing a person vocalizing a syllable and reported loss of habituation when a lag was inserted between the auditory and visual stimuli (Lewkowicz, 2010). The necessity of visemic analysis in speech acquisition is further congruent with the finding that preschoolers who are poor lip readers also have difficulty articulating speech (Desjardins et al., 1997). In some cases, this critical period for language acquisition appears at a later developmental stage. This pattern is exemplified by studies reporting that congenitally deaf children, shortly after being equipped with cochlear implants, perceived speech better when they were allowed to read lips than when they only heard speech or saw lip movements in isolation (Lachs et al., 2001; Bergeson et al., 2005).

### The AVS-ADS connection and the liberation from lip movement imitation

As the model suggests, the strengthening of the representations of calls in the ADS during *Hominan* evolution resulted in a brief period of vocal mimicry, limited to infancy, in which individuals acquired a long-term store for the vocal properties of calls (the phonological lexicon; in Figure 3C, top). However, in parallel with the encoding of phonological representations in the ADS, the repeated perception of a heard call during the mimicry period also resulted in the infant encoding an acoustic representation of the call through the sound recognition mechanism of the AVS. I therefore propose that due to simultaneous co-activation in both the AVS and ADS, connections formed between the acoustic-semantic representations in the AVS and their corresponding phonological (phonemic-visemic-praxic) representations in the ADS. In such a scenario, after an individual has passed the vocal mimicry period and has acquired the phonological lexicon, mimicry becomes purely auditory (i.e., independent of lip reading) because sound recognition in the AVS can now activate the corresponding phonemic-visemic-praxic representation in the ADS (Figure 3, middle). As the individual matures and the word is practiced regularly without the need to imitate lip movements, the visemic representation in the ADS weakens and the connections between the phonemic and praxic representations become more robust (Figure 3, bottom). This transition to purely auditory vocal mimicry (speech repetition) would have enabled the teaching or rehearsal of new words in the darkness of caves or during the night, when more time for bonding and practice was available.

Studies of brain-damaged patients with auditory perceptual deficits demonstrate the dependence of speech repetition in the ADS on sound recognition in the AVS. In a systemic comparison of such patients in the scientific literature (Poliva, 2014), 217 patients were identified as having loss of both sound comprehension and speech repetition (183 auditory agnosia patients and 34 cerebral deafness patients), but only 8 cases exhibited impaired sound comprehension but preserved speech repetition (word meaning deafness patients). If speech repetition deficit was due solely to ADS damage and speech comprehension deficit was exclusively the result of AVS damage, then word meaning deafness would have been significantly more common. Moreover, an intra-cortical electrical stimulation study demonstrated that stimulation in varying locations along the superior temporal gyrus-sulcus interfered with both speech repetition and comprehension, whereas stimulation in the MTG only interfered with speech comprehension (Boatman et al., 2000). These findings suggest that auditory agnosia is caused by damage in the region of the auditory cortex responsible for both speech comprehension and repetition, whereas word meaning deafness is caused by MTG impairment or disconnection of MTG from the auditory cortex. Additional support for the dependence of speech repetition in the ADS on sound recognition in the AVS is provided by electrical stimulation studies that localized auditory agnosia to the aSTG by showing that stimulating this region results in the transient loss of speech comprehension (Lachaux et al., 2007; Matsumoto et al., 2011; Roux et al., 2015); a passive listening fMRI study of an auditory agnosia patient with brainstem damage (i.e., intact cortex) reported, in addition to bilateral activation reduction in the aSTG, reduced activation in the left pSTG (Poliva et al., 2015). These findings imply that after a sound is recognized in the aSTG, the auditory information is transferred to the left pSTG (Figure 3 middle and bottom—arrow between **A** and **C**). Support for the view that the aSTG has a unique role in the recognition of syllables in speech repetition, in parallel to its role in the recognition of complete words, and in the transference of this acoustic information to the ADS has been provided by an intra-cortical recording study that recorded from both right and left superior temporal gyri while patients heard single words, and reported of two types of activation: activation for complete words, and activation that is selective for specific syllables/phonemes (Creutzfeldt et al., 1989). This activation pattern also remained the same when the patient was only hearing the word and when was repeating it. Evidence for a role of the aSTG in recognizing syllables and transferring this acoustic information to the pSTG is also provided by an fMRI study that compared the repetition of real words to the repetition of nonsense words composed of a repeated syllable (e.g., “tatata”) and to the naming of environmental sounds (Giraud and Price, 2001). The study revealed that there is stronger activation in the aSTG and pSTG when individuals repeat real words or syllables than when they name sounds. The study also showed stronger activation in the aSTG during the repetition of syllables than during the repetition of words (possible because pseudowords like ‘tatata’ require the recognition of 3 words), whereas the difference between words and syllables was notably smaller in the pSTG. Given that the aSTG processes both familiar syllables and real words, the hypothesis that auditory repetition is dependent on processing in the AVS prior to ADS processing is also provided by an fMRI study that instructed participants to rehearse and recall lists of 2–3 spoken words and reported that activation in the left superior temporal gyrus and sulcus preceded activation in the left Spt region (Buchsbaum et al., 2005). fMRI studies that reported of aSTG activation during the identification of discrete and meaningless syllables (Binder et al., 2004; Liebenthal, 2005; Ahveninen et al., 2006; Leff et al., 2009a; Woods et al., 2011) are also congruent with the role of this region in the transfer of acoustic-syllabic information to the pSTG. An evolutionary transition to purely auditory speech repetition through the development of aSTG-pSTG connections would also explain the ability of auditory agnosia patients to improve speech comprehension by watching lip movements (Buchman et al., 1986; Shindo et al., 1991). The present model suggests that, lacking intact processing in the aSTG, auditory agnosia patients resort to extracting meaning from spoken words through the more primitive speech repetition function of the ADS, which is dependent on the imitation of lip movements.

An alternative route for the transfer of information from the AVS to the ADS during speech repetition is via the direct connections between the semantic lexicon of the MTG and the phonological lexicon of the Spt-IPL region (Figure 3 middle and bottom—arrows between **B** and **D**) that are active during object naming (Edwards et al., 2010). However, based on reports of a dissociation between patients with impaired speech repetition but preserved object naming and patients with impaired object naming but preserved speech repetition (Hanley et al., 2004; Goldrick and Rapp, 2007), it is likely that acoustic-syllabic information travels to the phonological lexicon in separate pathways during speech repetition and during object naming. A study in which semantic dementia patients (MTG damage) and healthy controls were instructed to rehearse and recall lists of nonsense words and lists of both words and nonsense words reported that the healthy participants committed speech errors only when recalling the nonsense word lists, whereas the semantic dementia patients committed the same number of errors during recall of both lists (Hoffman et al., 2009). This study thus demonstrates the role of the aSTG-pSTG pathway in relaying acoustic information because it shows that without the MTG-Spt/IPL pathway, all words are treated as nonsense words during speech repetition. Additional evidence for the existence of both semantic and non-semantic routes to the phonological lexicon is provided in a study of aphasic patients who compared their performance on speech repetition tasks that required or did not require semantic processing (read words alone or in a coherent sentence; McCarthy and Warrington, 1984). The authors reported that aphasic patients with impaired speech repetition but preserved comprehension exhibited improved speech repetition when only semantic input was available, whereas an aphasic patient with impaired speech comprehension but preserved repetition exhibited improved speech repetition only when the word was isolated. A study also reported that aphasic patients with isolated deficit for repeating words have damage to the MTG whereas patients with isolated deficit for nonsense words have isolated damage to the Spt region (Baldo et al., 2012). These findings suggest that damage to the aSTG-pSTG-Spt/IPL pathway limits speech repetition to occurring solely via the MTG-IPL pathway, which relays only words that are encoded in the semantic lexicon. Damage to the aSTG-pSTG pathway could therefore correspond with the disorder deep dysphasia because these patients are unable to repeat nonsense words and produce semantic errors when instructed to repeat real words (Michel and Andreewsky, 1983; Metz-Lutz and Dahl, 1984; Dumahel and Poncet, 1986). Based on differences between brain-damaged patients with impaired repetition of nonsense words but preserved or impaired recall of verbal written material from working memory, it has been proposed that working memory for spoken words exists in two separate memory buffers (Jacquemot and Scott, 2006; Jacquemot et al., 2011). The input memory buffer is responsible for extracting sub-lexical information (e.g., syllables, phonemes) from the acoustic structure of the spoken word, and the output memory buffer (which corresponds with the speech production system) is responsible for sub-vocal rehearsal. The authors also argue that two pathways connect the two memory buffers: a path for repeating nonsense words and a path that passes through the semantic lexicon and facilitates the repetition or rehearsal of familiar words (Jacquemot and Scott, 2006). The input and output memory buffers thus correspond closely with the recognition of syllables and words in the aSTG and the speech production function of the Spt-IPL region.

### The ADS and the concatenation of syllables

In present-day humans, infants' first vocalizations are monosyllabic calls (baby coos) or bi-syllabic calls comprising repeated syllables (e.g., “mama”), and only at later developmental stages do they vocalize words with more syllables. This pattern implies that monosyllabic words are the building blocks of polysyllabic words. According to the present model, the evolutionary emergence of polysyllabic calls occurred only after *Hominans* acquired a monosyllabic lexicon. However, to delineate the evolutionary stages that led to the transition from monosyllabic to polysyllabic calls, we must first discuss the distinct roles of the AVS and the ADS in modern humans' processing of polysyllabic words.

**Figure 4:**
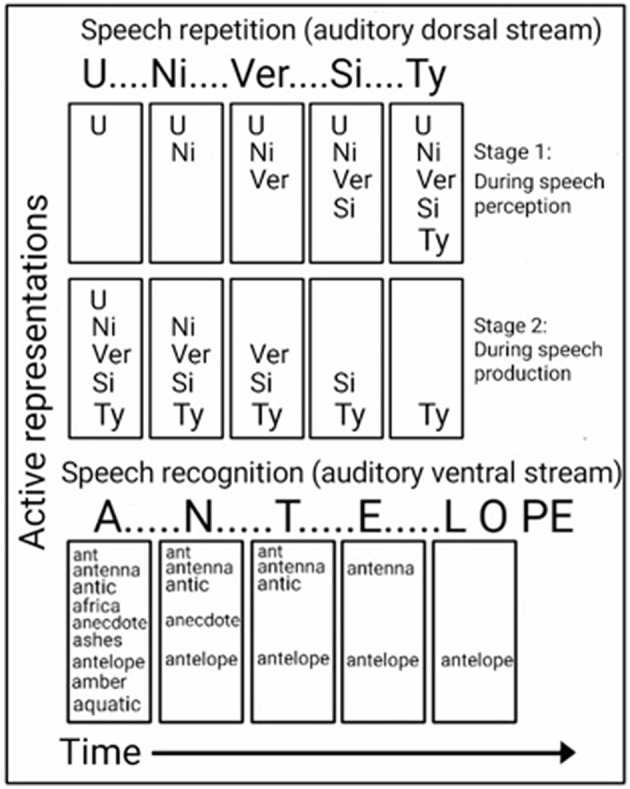
**Computational views of the auditory ventral and dorsal streams**. Top: In the ADS, repetition of a word (e.g., “UNIVERSITY”) is accomplished in two stages. While hearing the word (top), each syllable is encoded in order into a temporary memory buffer. Only after all syllables have been encoded (bottom), are the syllables extracted from the storage space (in the same order) to vocally produce the word. Bottom: In the AVS, a word (e.g., “ANTELOPE”) is recognized while being perceived. The first utterance (“A”) activates many possible matches, and the perception of each additional utterance further narrows the number of possibilities until only one match remains.

Insight into the computations performed in the ADS during speech repetition can be found in studies of brain-damaged patients, who were reported to exhibit better repetition for shorter words than longer words (Caramazza et al., 1986; Gandour et al., 1991; Franklin et al., 1996; Shallice et al., 2000; Nakakoshi, 2001; Jacquemot et al., 2011), and studies of healthy participants' who were better at recalling lists of short words than long words (Baddeley et al., 1984). This word length effect has long been advanced as evidence of two-stage processing during recall and repetition. In accordance with this model, syllables are first encoded, in order, in a temporary storage space (i.e., the phonological buffer; Figure 4, top). They are then extracted from the storage space and vocalized, in the same order, via the speech production system (i.e., the phonological loop; Baddeley et al., 1984, 2002). Evidence for the role of the ADS in the storage of syllables for speech production is provided by fMRI studies in which participants were instructed to covertly name objects, after which the number of syllables in the name was correlated with the activation strength in the ADS (pSTG, pSTS, Spt, IPL) (Okada et al., 2003; Shuster and Lemieux, 2005). Demonstrating that the ADS is also responsible for encoding the order of the syllables is an fMRI study that contrasted judgment of syllable order in nonsense words with syllable identification or identification of a speaker's gender and reported stronger activation in the IPL of the ADS (Moser et al., 2009; for additional order judgment studies implicating the IPL see: Marshuetz et al., 2006; Battelli et al., 2007).

Insight into the computations performed in the AVS during speech recognition can be found in a study of a word meaning deafness patient, who in addition to getting tested for word length with speech repetition, was tested for word length with speech recognition (Franklin et al., 1996). As expected, this patient was reported to exhibit better speech repetition for shorter words than for longer words (word length effect). However, in the speech recognition test, the patient performed better for longer words than for shorter words (reverse word length effect). Howard and Franklin (1988) also reported of a patient with better speech recognition of longer words. In contrast to the preference for shorter words during speech repetition, the preference for longer words during sound recognition (the reverse word length effect) is counter-intuitive because a long word (e.g., “ELEPHANT”) contains more information than a short word (e.g., “ANT”); therefore, the shorter word should be easier to perceive. An explanation for this superior recognition of longer words is provided by a model of word recognition (distributed cohort model), in which hearing the word's first syllable activates many candidate words (i.e., word-initial cohort), and hearing each additional utterance gradually narrows down the number of matches until only one remains (i.e., recognition point; Figure 4, bottom; Marslen-Wilson, 1987; Gaskell and Marslen-Wilson, 2002). This model is based on the finding that hearing an ambiguous incomplete word (i.e., end before the recognition point) primes all words that begin with that onset (e.g., the word “capt” primes both the words “captain” and “captive”; Marslen-Wilson, 1987), and that replacing the word onset, interferes with this priming effect (Marslen-Wilson, 1987; Marslen-Wilson and Zwitserlood, 1989). Demonstrating the activation of several words prior to the recognition point are EEG studies that presented participants with sentences, in which one word begins with a very probable meaning, but then switches to an unexpected, yet appropriate, word (Connolly and Phillips, 1994; van den Brink et al., 2001). For example, in the sentence “Phil put drops in his icicles” the listener predicts the last word to be “eyes” and not “icicles.” The researchers correlated the change of meaning in the middle of a word with an EEG component (ERP component N200; also called PMN). An EEG study that compared the hearing of words with an early recognition point with a late recognition point, further correlated the time of the recognition point with its own EEG component (ERP component N400; O'Rourke and Holcomb, 2002). Associating such computations with the AVS is evident by a study that recorded neural activation directly from the superior temporal gyri of both hemispheres (Creutzfeldt et al., 1989), and reported of rebound excitation after short, but not long, words. This initial inhibition could correspond with the initial word cohort. The authors further reported of neural excitation in response to hearing words that initiates only after the second or third syllable is perceived and of maintaining this excitation until the end of the perceived spoken word. This excitation could correspond with the recognition point.

Taken together, the findings presented in this section argues for different computations occurring in the AVS and ADS. In accordance with the computational model ascribed here for the AVS, the recognition of a word occurs in parallel to perceiving the word; therefore, the AVS does not entail calls to be segmented into syllables for optimal performance. The computational model ascribed here for the ADS, however, indicates that speech repetition is dependent on the ability to serially segment calls into discrete syllables; thus, it was the ADS that was modified through evolution to allow the repetition of polysyllabic calls.

### The ADS and phonological working memory

In an auditory working memory study, monkeys were trained to retain a sound in memory and to determine whether subsequently presented sounds were different from or identical to it (auditory delayed match to sample task with intermittent auditory interference; Scott et al., 2012). The authors observed that after each presentation of a non-matching sound, the difficulty of maintaining the acoustic properties of the first sample sound in working memory increased incrementally. This study thus showed that non-human primates (and, therefore, also our apian ancestors) experience difficulty maintaining sounds in memory. In contrast to the fleeting acoustic memory of non-human primates, humans easily hear, rehearse and then recall sounds, especially spoken words. An fMRI study that compared rehearsal and recall from working memory for tones and words further reported that both activated the ADS (Koelsch et al., 2009), indicating that it was the ADS that was modified during *Hominan* evolution, advancing from tone rehearsal to spoken word rehearsal. Based on the evidence presented in the previous section that the ADS stores the representations of syllables in memory during speech repetition, I argue that our enhanced working memory evolved for the purpose of repeating words with increasing numbers of syllables.

**Figure 5:**
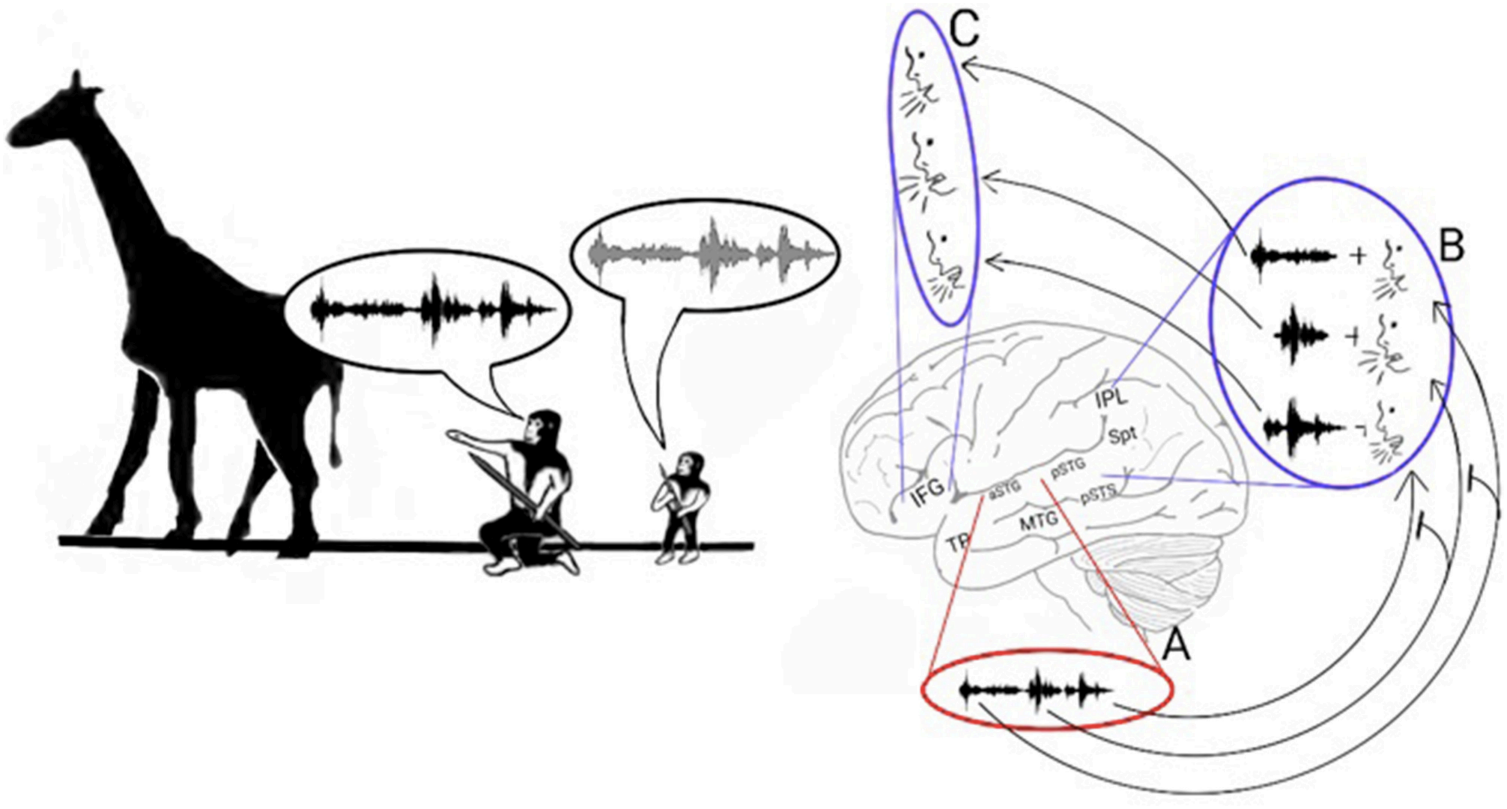
**Stage 3: The poly-syllabic lexicon and the enhancement of working memory**. Left: The model proposes that the transition from monosyllabic to polysyllabic repetition of calls was made possible by the development of a storage capacity in working memory. Speech repetition is initiated when the aSTG of the AVS perceives a call. As the aSTG-MTG extracts the call's meaning (not shown in figure), the aSTG also recognizes the individual syllables in the order in which they were perceived **(A)**. Each acoustic syllabic representation in the AVS activates its corresponding monosyllabic phonological representation in the pSTG-Spt-IPL regions of the ADS **(B)**. The Spt-IPL then activates the corresponding praxic representation in the IFG **(C)**. The storage capacity in working memory was made possible by the development of inhibitory connections in which each monosyllabic acoustic representation in the aSTG suppresses the phonological representation of the syllable that succeeds it in the pSTG-Spt-IPL (T-shaped arrows). Because of this process, the succeeding syllable is dis-inhibited and vocalized only after the present syllable has been vocalized.

In a previous Section (The AVS-ADS Connection and the Liberation from Lip Movement Imitation), I described a model of working memory that proposes a 3-step speech repetition process (Jacquemot and Scott, 2006). The acoustic structure of the word is decoded and stored in the input memory buffer. The syllables are then encoded in the output memory buffer, which is part of the speech production system. Finally, the syllables are extracted from the output memory buffer and vocalized in the order they were perceived. Within the present model, these findings suggest that the repetition of polysyllabic calls (Figure 5) occurs because, in addition to recognizing the whole word, the aSTG also recognizes its constituent syllables. This auditory recognition of syllables based on their order then activates, in the same order, the phonological representations of the calls in the pSTG-Spt-IPL region. Once the last phonological representation is activated, the syllables are vocalized in the same order via projections that target the praxic representations in the IFG. Because activation in the Spt-IPL region correlates with the number of syllables required for speech production (Okada et al., 2003; Shuster and Lemieux, 2005), a possible developmental change that enabled the transition to polysyllabic words is that the acoustic representations of syllables in the aSTG region acquired an inhibitory influence on the phonological representation of the succeeding syllable in the pSTG-Spt-IPL region (indicated by T-shaped arrows in Figure 5). This organization would result in the vocalization of each syllable, leading to the dis-inhibition and vocalization of the subsequent syllable, producing a chain reaction in which all syllables are vocalized in their correct order. This view is compatible with many working memory models that associate storage capacity in working memory with lateral inhibition (i.e., competitive queuing) and inhibition of vocal production (i.e., response suppression; see Hurlstone et al., 2013 for a review).

## Chunking and the emergence of sentences

**Figure 6:**
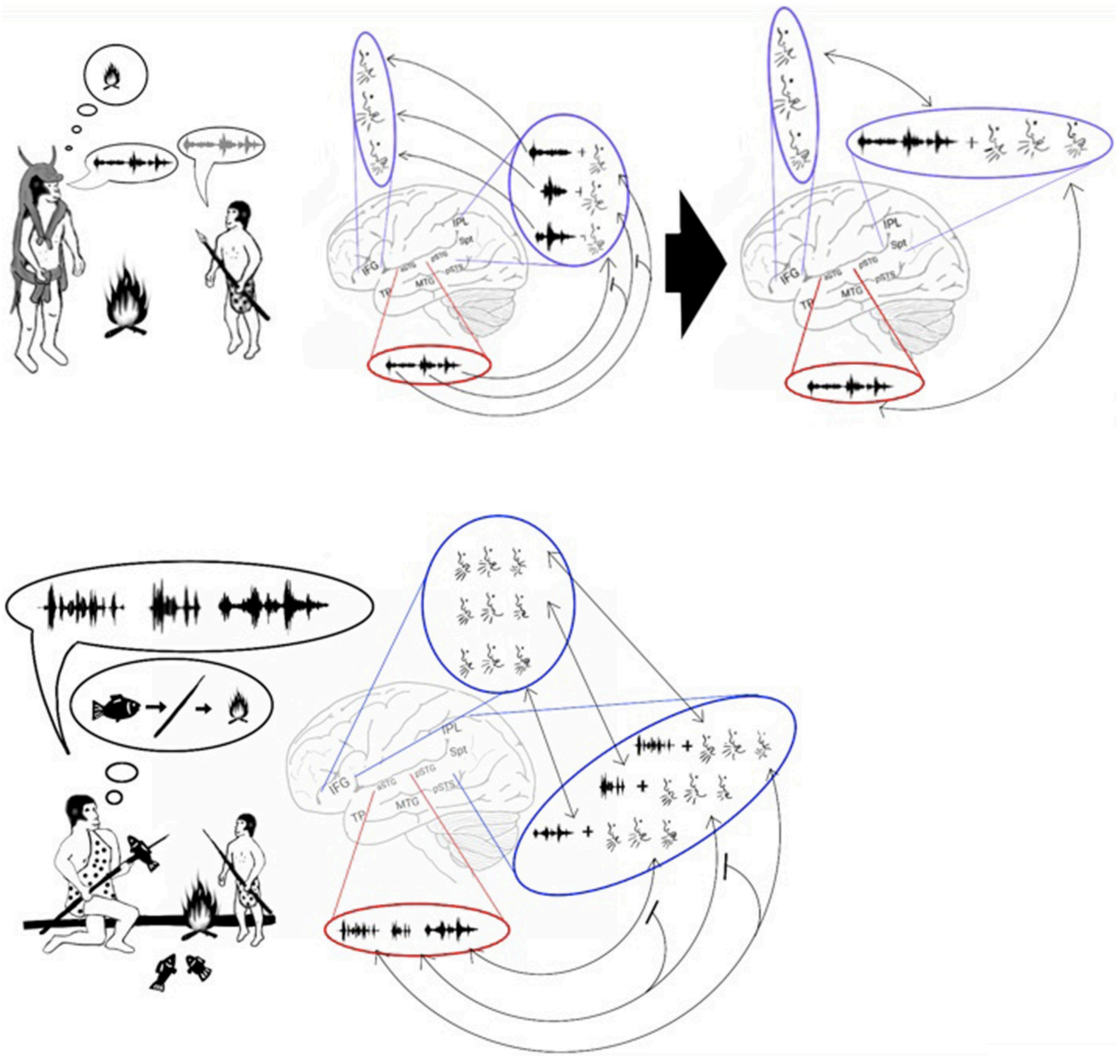
**Stage 4: Chunking and the emergence of sentences**. Top: The model proposes that, with the advent of chunking, constant rehearsal of a polysyllabic word (left) resulted in the word being encoded in the phonological lexicon as a single representation (right). Bottom: The encoding of a word as a single representation allowed individuals to vocalize and repeat lists of words. These lists could then be used to teach a sequence of actions. For example, the figure shows an adult teaching a child how to spear a fish and cook it over a fire by vocalizing 3 words: fish – spear – fire. The child can then repeat and thus memorize the sequence, and later he will be able to perform the sequence of actions on his own.

In the previous section, I proposed that the development of a storage capacity in working memory was sufficient to enable the maintenance in memory of more than one syllable at a time. Despite this development, however, working memory could maintain only one word at a time. This advance, therefore, does not explain the ability of our species to maintain a list of words in working memory. Based on the discovery of common features in both nonsense word repetition and word list recall (e.g., both have primacy and recency effects; Gupta, 2005; Gupta et al., 2005) and that aphasic patients with Spt-IPL damage are impaired in both tasks (Baldo et al., 2012), I argue that our ability to recall lists of words emerged from our ability to repeat lists of syllables. A transition from encoding syllables to words in working memory may have occurred because the associations between the syllabic representations strengthened until they began to be processed as a single representation (Figure 6, top). This process is called chunking (Miller, 1956). For example, modern humans engage in chunking when memorizing foreign words. Initially, each of these words is remembered as a list of meaningless syllables. After rehearsal, however, each of these words is remembered as a single unit. The chunking of syllables should therefore result with the ADS encoding phonological representations of words in addition to syllables. Indeed, cumulative evidence suggests that the ADS encodes both types of representations. For example, an fMRI study measuring activation changes across time reported that passively listening to words and nonsense words, but not reverse words, activates the ADS (pSTG, Spt, IPL). Furthermore, the temporal and spatial parameters of this activation are different when listening to nonsense words than when listening to real words, suggesting that real and nonsense words are processed by separate neural populations (Londei et al., 2010). Studies of word-meaning deafness patients who were capable of discriminating real words from nonsense words (lexical decision) also indicate that syllables and words are encoded separately in the ADS (Franklin et al., 1996; Hall and Riddoch, 1997; Bormann and Weiller, 2012). Indeed, functional imaging studies have correlated lexical decision performance with activation in the IPL of the ADS (Binder et al., 2003; Ischebeck et al., 2004; Xiao et al., 2005). The chunking of words should also result in each word representation being inter-connected with its corresponding syllabic representations. Evidence for the association between syllabic and word representations has been provided by fMRI studies reporting that ADS activation increases when participants read words with more phonological distractors (i.e., words that share syllables with a greater number of other words; Prabhakaran et al., 2006; Righi et al., 2010; Peramunage et al., 2011). Together, these findings suggest that the advent of chunking enabled our ancestors to maintain lists of words in memory. This development may have helped our ancestors teach and rehearse the sequences of words needed for hunting, tool making or cooking (Figure 6, bottom).

In the present model, working memory is treated as the temporary activation of the representations stored in long-term memory that are used for speech (phonological representations). Such sharing of resources between working memory and speech is evident by the finding that speaking during rehearsal results in a significant reduction in the number of items that can be recalled from working memory (articulatory suppression; Baddeley et al., 1984; Cowan, 2001). The involvement of the phonological lexicon in working memory is also evidenced by the tendency of individuals to make more errors when recalling words from a recently learned list of phonologically similar words than from a list of phonologically dissimilar words (the phonological similarity effect; Baddeley et al., 1984). A correlation has also been reported between speech production difficulty and the recall of the same words from working memory (Page et al., 2007; Acheson and MacDonald, 2009). Studies have also found that speech errors committed during reading are remarkably similar to speech errors made during the recall of recently learned, phonologically similar words from working memory (Caplan et al., 1992). Demonstrating the relationship of both speech production and working memory to the ADS is a study that induced magnetic interference in the pSTG and reported both speech errors while reading aloud and disturbance of the rehearsal in the working memory of nonsense words (Acheson et al., 2011; for a review of the role of the ADS in working memory, see Buchsbaum and D'Esposito, 2008). Patients with IPL damage have also been observed to exhibit both speech production errors and impaired working memory (Waters et al., 1992; Cohen and Bachoud-Lévi, 1995; Shallice et al., 2000; Shu et al., 2005). An fMRI multivariate analysis of visual working memory also detected similar cortical activation during a task that required participants to recall an item from long-term memory and while maintaining the same item in working memory (Lewis-Peacock and Postle, 2008). This finding shows that visual working memory, which likely operates in a similar manner to verbal working memory, also temporarily activates representations stored in long-term memory. Finally, the view that verbal working memory is the result of temporarily activating phonological representations in the ADS is compatible with recent models describing working memory as the combination of maintaining representations in the mechanism of attention in parallel to temporarily activating representations in long-term memory (Cowan, 2001; Oberauer, 2002; Unsworth and Engle, 2007; Barrouillet and Camos, 2012).

The most complex aspect of human communication is the production and comprehension of complex sentences. The ability to speak and understand sentences was likely derived from our ability to maintain lists of words in working memory. The strong relationship between working memory, sentence comprehension and the ADS was demonstrated in a study that compared the lesions and symptoms of 210 brain-damaged patients and reported a correlation between impaired working memory (low digit span), impaired sentence comprehension and damage surrounding the pSTG of the ADS (Leff et al., 2009b). Furthermore, a study (Heine and Kuteva, 2002) comparing the development of 350 grammatical rules from several contemporary languages argued that sentences in the parent language of these contemporary languages were composed of sequences of nouns (words for objects or events) and verbs (words for actions). For example, when describing the hunting of a rabbit, early *Hominans* may have communicated using the sentence “Rock—Throw—Rabbit” to express the command “throw the rock toward the rabbit.” This model is compatible with the present model and thus suggests that the emergence of chunking and, consequently, the ability to rehearse lists of words in working memory equipped *Hominans* with the necessary linguistic infrastructure for producing and comprehending grammatically simple sentences.

In the example sentence “Rock - Throw - Rabbit” the meaning of the sentence is dependent on the order of word presentation (i.e., the sentence “Rabbit - Throw - Rock” could communicate the meaning “throw the rabbit toward the rock”). For some aspects of grammar, however, meaning is not dependent on the presentation of a sequence of words in a specific order, as when describing the characteristics of nouns or verbs (adjectives and adverbs). For example, the meaning of the sentences “Rock - Throw - Slow - Rabbit” and “Rock - Throw - Rabbit - Slow” is the same (i.e., throw the rock toward the slow rabbit). Consistent with this view, adjectives are placed before a noun in some languages (e.g., English), whereas in others, the adjective follows the noun (e.g., Hebrew, French). A large body of research suggests that the ADS and AVS contribute differently to the processing of ordered and non-ordered (commutative) word sequences in sentences. Patients with damage to either the MTG or IPL have been reported to exhibit sentence comprehension difficulties; patients with MTG damage struggle to extract meaning and patients with IPL damage struggle to repeat sentences verbatim (Selnes et al., 1985; Martin et al., 1994; Bartha and Benke, 2003; Dronkers et al., 2004; Baldo et al., 2008; Magnusdottir et al., 2012). The role of the AVS in extracting the semantic properties of sentences has been demonstrated in functional imaging studies reporting stronger activation in the anterior MTG when proper sentences are contrasted with lists of words, sentences in a foreign or nonsense language, scrambled sentences, sentences with semantic or syntactic violations and sentence-like sequences of environmental sounds (Mazoyer et al., 1993; Humphries et al., 2001, 2005; Vandenberghe et al., 2002; Friederici et al., 2003; Xu et al., 2005; Rogalsky and Hickok, 2008; Pallier et al., 2011). One fMRI study in which participants were instructed to read a story further correlated activity in the anterior MTG with the amount of semantic and syntactic content each sentence contained (Brennan et al., 2012). An EEG study that contrasted cortical activity while reading sentences with and without syntactic violations in healthy participants and patients with MTG-TP damage, concluded that the MTG-TP in both hemispheres participate in the automatic (rule based) stage of syntactic analysis (ELAN component), and that the left MTG-TP is also involved in a later controlled stage of syntax analysis (P600 component; Kotz et al., 2003). In contrast to the role of the AVS in extracting meaning from sentences, evidence indicates that the ADS is involved in the encoding of words and clauses in working memory. Functional imaging studies of healthy participants have shown that when readers need to re-order the clauses or words in a sentence to extract its meaning (syntactic transformations), activation increases primarily in ADS regions (pSTG, pSTS, IPL, IFG; Just et al., 1996; Caplan et al., 2002; Ben-Shachar et al., 2003, 2004; Bornkessel et al., 2005; Fiebach and Schubotz, 2006). A recent model developed by Bornkessel-Schlesewsky et al. (2015) proposes, on the basis of this division of labor between the two processing streams, that the AVS extends its role in forming multi-modal semantic representations to sentence comprehension by performing the commutative integration of words in a sentence (e.g., merging the words “slow” and “rabbit” to form the combined concept of a slow rabbit). In contrast to the commutative role of the AVS in sentence comprehension, the authors argue that the ADS contributes to sentence comprehension by processing the order of words in sentences. The convergence of the two pathways in the IFG then enables the comparison of the information from both processing streams and the comprehension of the sentence. In the grammatical evolution model proposed by Heine and Kuteva (2002), the authors argue that adjectives and adverbs (the commutative elements of the sentence) evolved from nouns and verbs (and that the remaining grammatical terms are further derivations of verbs and adverbs). In accordance with that model, and with the model of Bornkessel-Schlesewsky et al. (2015), the present model suggests that when word lists of verbs and nouns began to be used as preliminary sentences, the necessary infrastructure for enriching these sentences with adjectives and adverbs (and, later, also other grammatical terms) was already in place.

## Concluding remarks and future research

In this manuscript, I propose a novel, plausible evolutionary process that explains the transition from basic vocal control to complex language characterized by rudimentary grammar. I argue that once *Hominans* acquired volitional control over the vocal apparatus and were capable of naming objects, the primary process by which language became incrementally more complex was the gradual enhancement of the ability to store heard vocalizations in temporary memory, which was utilized for learning novel vocalizations via mimicry/repetition. In contrast to most models of language evolution, which are based on research of fossils, contemporary languages or human behavior, the present model is based directly on knowledge, accumulated in the past two decades, of sound, speech and language processing in the brain. Importantly, this is the first language evolution model to propose an explanation of the varied functional co-localization of the ADS. This model is also parsimonious because it provides a plausible explanation for the emergence of non-language human characteristics, such as our pronounced lips, the vocal mimicry of young children and our enhanced working memory. This model is also validated by its ability to explain findings from brain research that, so far, have been considered anecdotal (e.g., semantic paraphasia in deep dysphasia patients, the reverse word length effect observed when participants with word meaning deafness perform sound recognition tasks, the remarkable preservation of lip-speech integration in auditory agnosia patients).

Although many studies support the present model, some additional research is needed. In the first stage of the model, I associate visemic analysis with the ADS. Although one MEG study demonstrated that visemic analysis occurs in the ADS (Nishitani and Hari, 2002), more studies are needed. Future studies should also explore the relationship between visemic analysis and the phonological lexicon. For example, researchers could test whether seeing the lip movements associated with a word primes words with a similar phonological structure. Conversely, researchers could test whether hearing or reading a word improves lip-reading of words that are enunciated using similar lip-movements. In the second stage, I propose that mimicry, which is dependent on the imitation of lip movements, was restricted to infancy. Such dependence of speech development on lip-speech integration can be tested in a future study that explores whether congenitally blind adults speak with a different range of lip-movements than adults with acquired blindness and whether congenitally deaf adults speak with a different range of phonemes than adults with acquired deafness. The hypothesis that speech became auditory as the connections between the aSTG and pSTG developed also needs to be corroborated by future research. If such connections are critical for speech repetition, intra-cortical electrical stimulation of the aSTG should impair repetition and comprehension and result in reduced activation in the pSTG. In the third stage of language evolution, partially based on recordings from the right and left superior temporal gyri during speech comprehension and repetition (Creutzfeldt et al., 1989), I propose that only the ADS is involved in the segmentation of calls into syllables. This study, thus needs to be replicated. Supporting the role of the AVS in recognizing spoken words in parallel to hearing them is also a case study of a brain-damaged patient who exhibited the word length effect during speech repetition and the reverse word length effect during speech comprehension (Franklin et al., 1996). The effect of syllabic length on speech recognition and repetition should also be replicated in additional patients. Based on the correlation between strength of activation signal in the Spt-IPL region and syllabic length (Okada et al., 2003; Shuster and Lemieux, 2005), I also propose that working memory emerged due to the development of inhibitory connections between the acoustic representations in the aSTG and the phonological representations in the pSTG-Spt-IPL region. However, because fMRI studies cannot determine whether activation is caused by inhibitory or excitatory afferents, this experimental paradigm needs to be replicated with direct recording from the cortex in the pSTG-Spt-IPL region. In the final stage, I propose that once *Hominans* were able to encode a string of syllables as a single lexical representation in the phonological lexicon, they became capable of rehearsing and communicating word lists. Although the ADS has been shown to encode both syllabic and lexical phonological representations, little is known about the neuroanatomical correlates of chunking. To test whether the ADS is directly involved in chunking syllables as words, fMRI can be applied to participants as they attempt to rehearse long strings of syllables in which some syllabic combinations appear with high frequency. I predict that as the participants recall longer strings of syllables (due to the chunking of frequent syllabic combinations), more activation will be observed in the ADS.

In conclusion, I believe that the present model has the potential to contribute to the scientific community on several levels. First goal of the present model is to demonstrate to scholars outside the field of neuroscience that sufficient knowledge has been obtained from brain research in the last two decades to justify its use as a tool in the development of new models of language evolution. A second goal of the paper is to inspire more neuroscientists to investigate the origins of language. Finally, with this paper I hope to provide the scientific community with new lens for viewing language processing in the brain.

## Author contributions

The author confirms being the sole contributor of this work and approved it for publication.

### Conflict of interest statement

The author declares that the research was conducted in the absence of any commercial or financial relationships that could be construed as a potential conflict of interest.
